# The Nexus between Pollution and Obesity and the Magnifying Role of Media Consumption: International Evidence from GMM Systems Estimates

**DOI:** 10.3390/ijerph191610260

**Published:** 2022-08-18

**Authors:** Cristiana Tudor

**Affiliations:** International Business and Economics Department, The Bucharest University of Economic Studies, 010374 Bucharest, Romania; cristiana.tudor@net.ase.ro

**Keywords:** obesity, pollution, media consumption, interaction, economic development, system-GMM

## Abstract

The aim of this paper is to uncover the associations between air pollution, media consumption, and the prevalence of obesity. Based on data availability, this study draws on an unbalanced panel of 28 countries and develops and extracts relationships through robust System-General Method of Moments (Sys-GMM) estimators that account for the dynamic nature and high persistence of the variables of interest. In light of previous findings, economic development, trade openness, and government consumption are included as controls in the dynamic panel models. The estimation results consistently indicate that pollution is a strong determinant of obesity, a link that remains robust through the alternative proxies for pollution (i.e., total greenhouse gas emissions (GHG) and carbon (CO_2_) intensity of energy generation). However, CO_2_ intensity shows the strongest association with obesity. Furthermore, the findings indicate that media consumption is an independent and significant driver of obesity, whilst its inclusion among regressors further magnifies the impact and significance of the pollution factor. Moreover, the combined effect of media consumption and pollution significantly contributes to spurring obesity in all model specifications. Thus, a vicious cycle emerges between air pollution, media consumption, and obesity, with synergistic detrimental health effects. The current findings highlight the importance of continuing and consistent efforts to mitigate pollution and reach related low-carbon policy targets. Moreover, for the sustainable reduction and prevention of obesity, these efforts should be complemented by policy interventions and public campaigns aimed at “healthy” media consumption, such as encouraging regular physical exercise and healthy nutrition.

## 1. Introduction

Air pollution is now regarded as the world’s most serious environmental health concern, responsible for 7 million deaths worldwide each year ([1,2]). Moreover, recent evidence highlights that chronic exposure to air pollution can impact every organ in the human body, complicating and increasing pre-existing health disorders [3]. As such, pollution is acknowledged as a significant driver for mortality and disease [4]. Furthermore, statistics disturbingly show that more than 99% of the global population lives in areas with air pollution levels that surpass the World Health Organization limits, as of 2019 [5,6].

**Figure 1:**
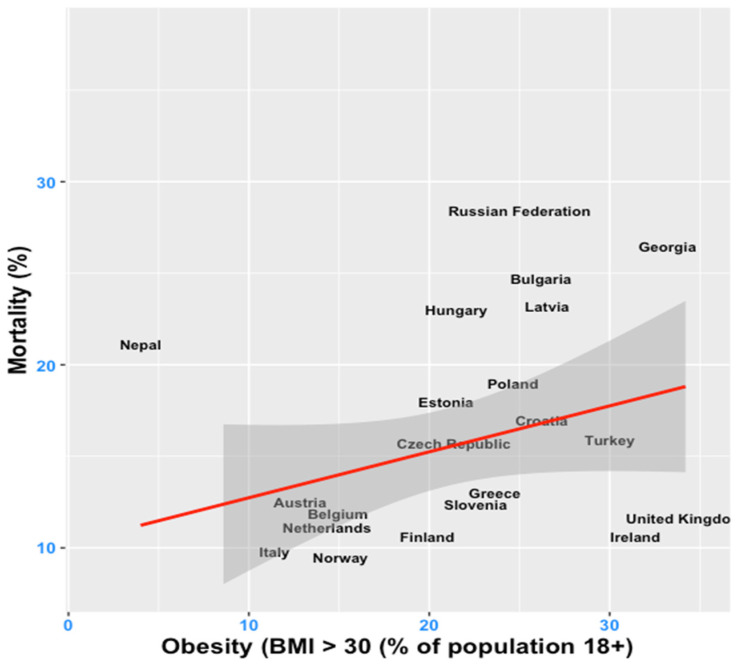
Obesity (percentage of population with a body mass index (BMI) > 30% in all adult population) and mortality, most recent year of available data per country. The red line represents the fitted linear regression line, while the dark grey area displays the 95% confidence interval for predictions from a linear model. Source of data: World Bank’s Development Indicators (WDI) database.

Concurrently, obesity remains a serious public health concern [7], often regarded as a worldwide epidemic [8,9,10,11], given estimations that indicate 25% of the world’s population is overweight, among which a third suffers from obesity [12]. The rate of early mortality due to a high body mass index (BMI) increased between 1990 and 2015 from 41.9 to 53.7 per 100,000 individuals, whereas a high BMI caused four million deaths globally over the same period, among which 60% of individuals had obesity and, in the majority of cases, died as a result of cardiovascular diseases [13]. The positive link between an increased risk of death from cardiovascular disease and an unhealthy body weight has been repeatedly documented [14,15,16]. This relationship also emerges from our study sample, with Figure 1 reflecting a clear positive association between the prevalence of obesity and mortality at the world level.

Additionally, the link between diabetes and weight gain has also been established [17]. Moreover, empirical evidence also confirms that there is a significant association between obesity and increased risk for certain cancers [18]. Furthermore, an additional non-trivial issue is that obesity and associated comorbidities may have a significant impact on healthcare expenditures [19], with estimates indicating relative economic burdens ranging from 0.09% to 0.61% of gross domestic product (GDP) at the country level [20].

Considering the above causal relationships, it is critical to identify causes of unhealthy body weight and develop strategies for long-term reduction and prevention.

Specifically, air pollution can cause an unhealthy body weight by causing metabolic dysfunction, the emergence of chronic disease, and the disruption of regular physical exercise [21]. However, the literature investigating the transmission channels between the two paramount factors impacting world population health remains surprisingly limited. In this narrow literature, [22] showed that traffic-related air pollution was positively and significantly connected with an increase in body mass index and obesity in children. [23] confirmed the association between air pollutants, obesity, and diabetes. More recently, [24] also documented that early-life exposure to air pollution is positively linked to an increase in the risk of developing overweight and obesity in childhood. In turn, [25] offered a potential transmission channel by arguing that air pollution is positively linked to sedentary behaviors. This is further reinforced by [26], who agreed that behavioral channels can explain the link between pollution and obesity, as air pollution may either decrease physical activity or may prevent people from engaging in physical activity. Additionally, [27] revealed another mechanism, as they concluded that thyroid function parameters, which in turn can cause obesity, are linked to exposure to climate and air pollution.

**Figure 2:**
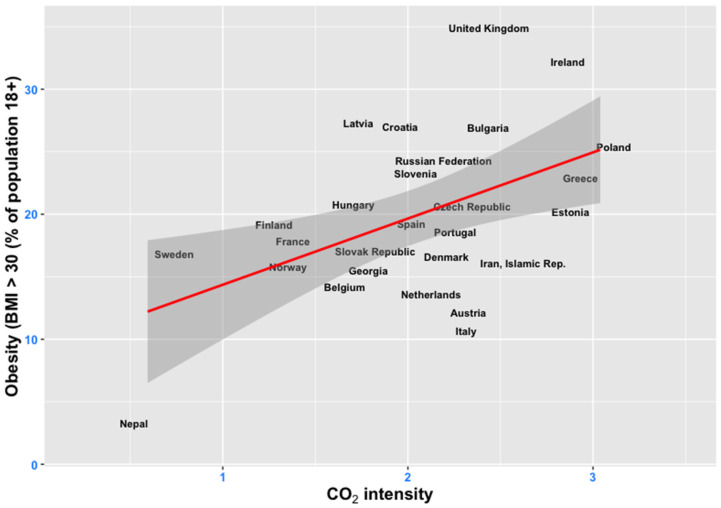
CO_2_ intensity (kg per kg of oil equivalent energy use) and obesity (percentage of population with a BMI > 30% in all adult population) for most recent year of available data per country. The red line represents the fitted linear regression line, while the dark grey area displays the 95% confidence interval for predictions from a linear model. Source of data: World Bank’s Development Indicators (WDI) database.

Our study contributes to extending this area of research by exploring the relationship between alternative pollution factors and obesity for a global panel of countries, and by employing a robust mix of control variables. A preliminary assessment of the link between pollution and obesity based on our data sample indicates a positive relationship between the two main variables of interest (Figure 2).

Moreover, we build on previous studies that conclude that increased media consumption is a driver for a sedentary lifestyle ([28,29]). In particular, there are well-established links between using the Internet for leisure and engaging in sedentary behavior in both children and adults ([30,31,32,33]). In this context, it can be reasonably assumed that increased media consumption, through the promotion of sedentary habits, raises the risk of obesity. We further link this hypothesis with the documented positive association between pollution and unhealthy body weight. We thus hypothesize that the combined effect of media consumption and pollution on obesity through behavioral channels such as physical inactivity can be synergistic. Consequently, another novelty of the current research relies upon the introduction of an interaction factor including a pollution proxy and a media consumption proxy, thus investigating whether increased media consumption and concurrent pollution exposure magnify this health problem for the sample of countries in this study.

Furthermore, in deciding on the control group, we consider previous evidence that links lower socio-economic status (SES) to increased exposure to air pollution [2], as well as previous findings that document positive associations between lower SES, high media consumption, and prevalence of overweight and obesity ([34,35,36]). Additionally, government consumption and globalization (trade openness) are expected to covary with the focal independent variables in light of previous results (among others, [37,38,39,40]), and are thus used as control or confounding variables [41].

This paper adds to the extant literature in several ways. First, it explores the relationship between pollution and obesity based on a global (albeit narrow, due to data unavailability) panel of countries by employing alternative factors to proxy for pollution and introducing a relevant mix of explanatory variables that has not been used before. Second, it proposes an interaction factor to assess a link that has not been investigated thus far, although indications point toward its relevancy, i.e., the combined effect of pollution and media consumption on obesity. To achieve its goals, this study employs the Generalized Method of Moment (GMM) for alternative model architecture estimation. Thus far, the GMM is acknowledged as the strongest technique for investigating dynamic interactions between variables [42]. Of note, the estimator caries the additional advantage of eliminating the need for distributional assumptions and resolving significant issues with panel data estimations [43].

The results provide updated evidence to highlight that obesity is persistent and is highly impacted by pollution (additionally, the relationship remains robust against alternative proxies). Furthermore, the findings indicate that media consumption is an independent significant driver for obesity, its combined effect with pollution contributes to significantly increasing obesity, and its inclusion as an independent factor further magnifies the effect of pollution. These findings have important policy implications, indicating that positive externalities emerge from global efforts to decarbonize economies, as well as pinpointing the importance of healthy media consumption, for whose attainment policy interventions and education campaigns are needed. The interplay between the two strategies can achieve sustainable low-carbon development and sustainable obesity reduction and prevention.

## 2. Material and Method

### 2.1. Data and Variables

The data for all variables in this study were collected from the World Development Indicators (WDI) database of the World Bank.

Pollution was the main explanatory variable in our analysis of obesity occurrence in a country, which was in turn represented by the prevalence of adult obesity, i.e., the percentage of adults ages 18 and over with a body mass index (BMI) of 30 kg/m^2^ or higher (WDI code: SH.STA.OB18.MA.ZS). This research used two alternative indicators to proxy the environmental pollution of a country. First, we employed one of the most commonly encountered proxies for pollution, i.e., total greenhouse gas emissions (GHG) ([44,45]). Second, we used the intensity of pollutant emissions, an indicator that is often used to compare the environmental impact of various fuels or activities. The ratio of carbon dioxide per unit of energy, or the amount of carbon dioxide emitted as a result of using one unit of energy in production, is known as carbon intensity, and it is often employed as a proxy for environmental (under)performance [46].

Furthermore, data for the following explanatory factors were also extracted: the percentage of Internet users in the total population (as a proxy for media consumption), GDP per capita as a proxy for the average socio-economic characteristics of the population, government final consumption expenditure, and trade openness.

Table 1 centralizes the variables employed in the empirical investigations, showing abbreviations, variables’ codes in the WDI database, and description.

The final data sample in this research was provided due to the availability of data and includes all countries with available data for the variables of interest for at least three years, which allows for the estimation of dynamic panel models. Consequently, we constructed an unbalanced panel spanning 16 years (i.e., 2000–2015) for 28 countries (see Table 2 for final countries list).

### 2.2. Exploratory Data Analysis

**Figure 3:**
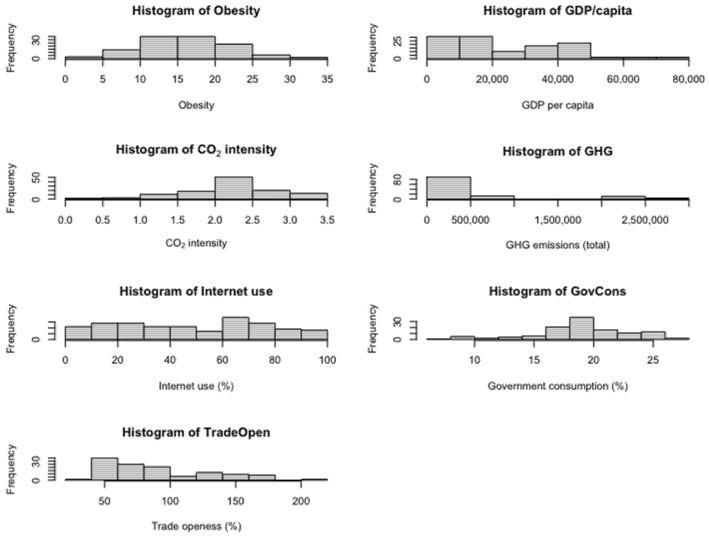
Histograms for the variables of interest.

One of the most relevant exploratory instruments is histograms of variables, which are presented in Figure 3. The distribution of the obesity variable is closest to normality, indicating that its mean value does not deviate much from its median. Interestingly, the distribution of CO_2_ intensity is similar to the distribution of government consumption, suggesting an association between public spending and environmental underperformance.

Further, descriptive statistics for the full sample are centralized in Table 3. The countries in the study sample register a mean level of 16.11 percent for the prevalence of obesity in the adult population, with a high range between a minimum level of 1.70 percent and a maximum of 34.20 percent. The exposure to media, as proxied by the percentage of Internet users, shows the highest variation within the sample, ranging from a minimum level of 0.38 percent to a maximum of 96.81 percent. The economic development, as proxied by the GDP per capita, also presents a high range, spanning from a minimum of USD 585.41 to a maximum of USD 74,355.52. The two variables might contain similar information that will emerge in estimations.

**Figure 4:**
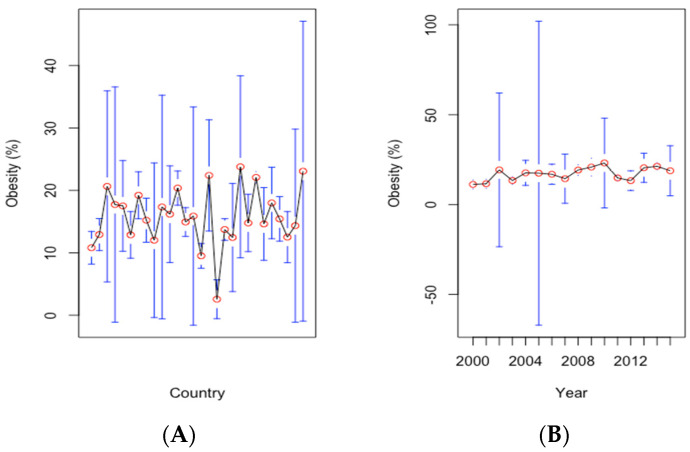
Mean prevalence of obesity (%) by country, including the confidence intervals (Panel **A**). The evolution of mean prevalence of obesity from 2000 to 2015, with confidence intervals (Panel **B**). Source of data: World Bank’s Development Indicators (WDI) database.

Additionally, we noticed a high heterogeneity of obesity prevalence across countries, whereas its time trend is rather smooth through the analysis period, suggesting that whereas time effects are not present, country effects should be considered for robust estimations (Figure 4). 

Finally, the correlogram of all variables is presented in Table 4, indicating on one hand that regressors in the model are free from severe collinearity, and additionally that there is a positive association between obesity and pollution. Of note, although some degree of collinearity among predictors is intrinsic, I argue that variables do not suffer from severe multicolinearity (i.e., correlation > 0.8) [47] that would affect estimation results.

### 2.3. Method

This section describes the general form of the model(s), presents relevant details on dynamic panel data estimation, and establishes the main hypotheses to be tested.

Firstly, to smoothen the data and generate more consistent findings ([47]), all variables were converted to their natural logarithm (ln) form. Consequently, the main relationship of interest based on the previous discussion is given by Equation (1), where all variables enter in their natural logarithm form:

(1) $$OB_{it}=\gamma OBES_{i,t-1}+\beta_{1}POLLUT_{it}+\beta {X^{\prime }}_{it}+\varepsilon_{it}$$

where *OBES_it_* is the current level for the obesity indicator in country *i*, *POLLUT* is the time-varying pollution measure in country *i* (proxied alternatively by total GHG emissions in the main models and also by CO_2_ intensity in further robustness checks), and *X* is a vector of control variables including the GDP per capita, trade openness, and government consumption. Lastly, *ε* denotes the error terms, the subscript *i* (*i* = 1, …, n) denotes the country *i* in the data sample, N = 28, and *t* (*t* = 1, …, T) indicates the time period, such that T = 16. 

A second relationship of interest investigates the potential magnifying effect that concomitant exposure to media (proxied by the percentage of Internet users) might have on the obesity prevalence among the population, such that:

(2) $$OBES_{it}=\gamma OBES_{i,t-1}+\beta_{1}POLLUT_{it}+\beta_{2}(POLLUT_{it}*Media_{it})+\beta {X^{\prime }}_{it}+\varepsilon_{it}$$

The interaction factor that we propose in this study constitutes a novelty, making for a relevant contribution to the existing literature. It relies on previous findings that link pollution and obesity, and, on the other hand, media consumption and obesity through the same transmission mechanism based on behavioral traits, such as a sedentary lifestyle.

The following hypotheses are tested via robust dynamic panel models: *β*_1_ in Equation (1) and *β*_1_ and *β*_2_ in Equation (2) are significant and positive. The impact of control variables, especially the link between income and obesity, is also of interest, serving for comparative purposes to previous findings. Based on empirical results, we expect a negative relationship between the level of economic development and obesity, although this link could depend on the sample of countries included in the investigation.

The presence of the lagged dependent variable on the right side of the equation in both Equations (1) and (2) characterizes a dynamic panel model [48], which raises various econometric issues in estimation that must be properly addressed for reliable inference ([42,49]).

Consequently, the System Generalized Method of Moments (Sys-GMM) estimator proposed by [50,51] is employed in this study to conduct the empirical investigation of the primary impact factors for obesity in the sample of 28 countries. The advantages of the Sys-GMM estimator are non-trivial. First, its alternatives, i.e., OLS and the Within estimators, are known to derive biased and inconsistent estimates in a dynamic context ([52,53]). The bias of these two approaches is eliminated by the system GMM estimator. Moreover, the Sys-GMM estimator also overcomes the issues of fixed effects and endogeneity of regressors, in addition to solving the problem of autocorrelation within individuals ([43,54]). Consequently, Sys-GMM not only provides consistent estimates [55], but it also brings significant efficiency gains in dynamic panel estimations ([56,57]). Not in the least, in comparison to the Difference GMM estimator developed by [58], the Sys-GMM allows for more instruments through an additional assumption under which the first differences of instruments are uncorrelated with fixed effects, thus emerging as a more efficient estimator ([49]). Consequently, the system GMM estimator combines two sets of equations and thus adds the level form moment conditions to the difference form moment conditions, hence reducing biases of the difference-GMM estimators [59].

The following dynamic panel equation estimates model (1) through a dynamic system-GMM analysis:

(3) $$LnOBES_{it}=\gamma Ln{\left(OBES\right)}_{it-1}+\beta_{1}LnPOLLUT_{it}+\beta_{2}LnGDP_{it}+\beta_{3}LnGCons_{it}+\beta_{4}LnTradeOpen_{it}+\mu_{i}+\phi_{t}+\varepsilon_{it}$$

*i* = 1,..., 28 and *t* = 2000,..., 2015, where the dependent variable representing obesity is explained by its own lagged value, two alternative proxies for pollution, the interaction factor between pollution and media consumption and other relevant controls, including the GDP per capita, trade openness, and government consumption, while $\mu_{i}$ stands for fixed country specific effects, $\phi_{t}$ represents time effects and $\varepsilon_{it}$ is an error term with zero mean.

Similarly, the model specified in Equation (2) takes the following extended form in the Sys-GMM estimation, where different mixes of regressors are alternatively tested:

(4) $$LnOBES_{it}=\gamma Ln{\left(OBES\right)}_{it-1}+\beta_{1}LnPOLLUT_{it}+\beta_{2}LnPOLLUT_{it}*LnMedia_{it}+\beta_{3}LnGDP_{it}+\beta_{4}LnGCons_{it}+\beta_{5}LnTradeOpen_{it}+\mu_{i}+\phi_{t}+\varepsilon_{it}$$

It must also be mentioned that the asymptotic efficiency benefits brought about by the Sys-GMM estimator’s added orthogonality assumptions produce a proliferation of instruments that leads to a finite sample bias. As such, as per [60] and similar to the approach of [61], we used only two lags for both the difference and system GMM estimators, and consequently specified a collapsed instrument matrix and robust coefficient covariance matrices proposed by [62] (i.e., in the estimation of the “pgmm” function within R’s “plm” package). Within the same function, the “Sys-GMM” estimator is obtained using transformation = “ld” for level and difference. All relevant details for estimating GMM models in R are found in the “plm” package description, which was developed by [63]. 

Finally, as GMM estimators are consistent if there is no second-order serial correlation for the idiosyncratic errors of the first-differenced equation, we tested for any remaining serial correlation by using the Arellano–Bond (AR) test. Furthermore, to ensure that the validity of instruments assumption is valid, overidentification was also tested by Sargan (1958)’s test of over-identifying restrictions [64], further developed by [65]. 

**Figure 5:**
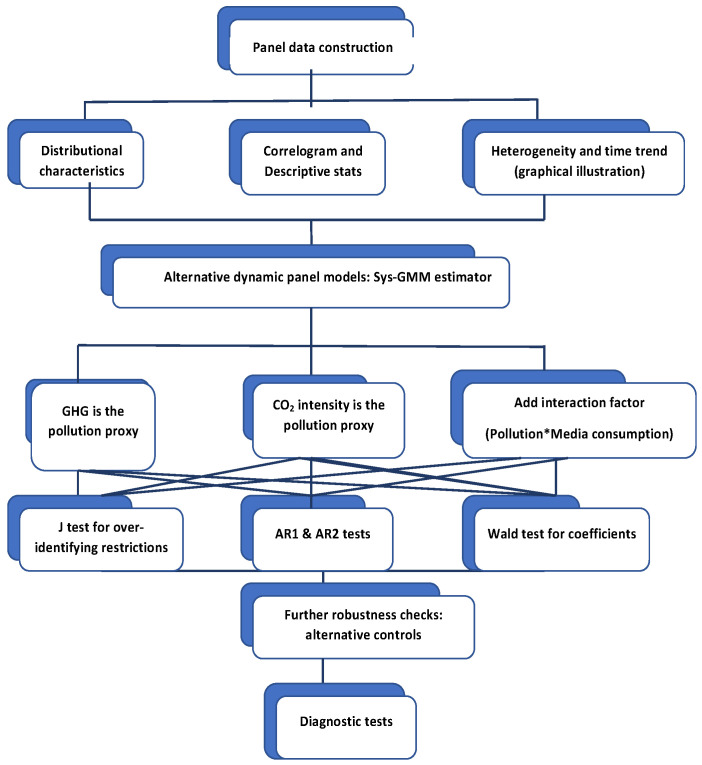
Overview of the implemented method. AR1 and AR2 represent the Arellano–Bond tests for first- and second-order autocorrelation in the idiosyncratic errors of the first-differenced equation.

Figure 5 provides an overview of the method and the specific steps taken to implement it. R software was employed for the overall method implementation, including all estimations.

## 3. Results

Of note, the two-step GMM estimators can be seriously biased downwards in finite samples ([51,66]). In this respect, only one-step System GMM estimates are reported in this section (similar to [49,67,68]), although two-step estimations were also performed. Additionally, we report results from seven alternative model specifications (i.e., M(1) to M(7)) out of many specifications that were estimated, underlining that the main relationships of interest remain significant throughout the specifications. Additionally, as mentioned earlier, the robustness of the System GMM estimators depends both on the assumption that the error term does not have a serial correlation problem and on the validity of instruments. Consequently, these assumptions are verified through the Arellano–Bond test for no serial correlation in the error terms and the Hansen/Sargan J test for the validity of the instruments, which are reported in the bottom rows of all results tables. Moreover, the results tables also include the Wald test of slope coefficients being zero jointly ([69]). The row for the J-test reports the *p*-values for the null hypothesis of the validity of the overidentifying restrictions, whereas the *p*-values reported for AR1 and AR2 are the *p*-values for first- and second-order autocorrelation in the first-differenced residuals equation. In all cases, diagnostic tests confirm that all models are properly specified, as the J test never rejects the null hypothesis of instrument validity, the Wald test always rejects the null hypothesis that slope coefficients are simultaneously equal to zero, and the AR2 test consistently confirms that there is no second-order auto-correlation in the differenced residuals.

### 3.1. The Relationship between Pollution and Obesity

Table 5 reports the baseline results of the estimation of Equation (3) with the System GMM estimator over the period of 2000–2015 for the unbalanced panel of 28 countries, where GDP per capita is a control variable in both models, and the GHG emissions are the first proxy for pollution (M(1)), while subsequently the CO_2_ intensity is introduced for the same task in M(2).

An analysis of the persistence of the obesity indicator for the countries in the sample confirms the a priori assumption that is based on previous empirical evidence, namely that higher obesity in the previous period contributes to obesity in the current period. The autocorrelation parameter is statistically significant and of higher magnitude in the second model specification (i.e., M(2)) when the CO_2_ intensity is used as a proxy for pollution, such that a 1% increase in the lagged obesity factor increases obesity in the current period by about 0.44%.

Moreover, GDP per capita has a positive and significant effect on obesity in M(1) when GHG emissions are employed as a proxy for pollution, but loose statistical significance when CO_2_ fulfills this task in M(2).

Most importantly, the coefficients of the pollution factor are positive and very significant for both model specifications, with the magnitude being higher for CO_2_ intensity (point estimate of 0.916) than for GHG emissions (point estimate of 0.088). Thus, a 1% increase in carbon dioxide intensity brings an increase in obesity of about 0.92%.

### 3.2. The Influence of the Interaction Factor

Table 6 reports the results of further System GMM estimates for Equation (4), where GHG in M(3) and CO_2_ intensity in M(4) are pollution proxies, GDP per capita is again a regressor in both models, and the interaction factor is introduced as the main impact factor.

The main coefficients of interest remain those corresponding to the pollution factor (GHG/CO_2_ intensity) and the interaction factor of the pollution and media consumption variables. As hypothesized, these coefficients are positive and very significant for both model specifications. This implies that pollution is an explanatory factor for obesity and additionally, media consumption is a promoter of obesity for the sample of countries in this study. Specifically, the M(3) model specification includes GHG as a pollution proxy, which is included both as an independent factor and in combination with media consumption (i.e., Internet use) within the interaction factor. The results indicate that the interaction factor increases the impact of the GHG factor from 0.088 to 0.131, whereas the interaction factor itself has a statistically significant impact with a point estimate for its coefficient of 0.03. Similarly, M(4) employs CO_2_ intensity both by itself and within the interaction factor. The interaction factor has a higher coefficient in M(4), equal to 0.06, whereas the addition of the factor contributes to substantially magnifying the impact of the pollution factor from 0.916 to 1.444. As such, a 1% increase in CO_2_ intensity brings an increase in obesity of about 1.44% when the interaction between pollution and media consumption is also included.

In conclusion, adding the interaction term changed the values of β1. The effect of pollution on obesity is now 0.131 + 0.03 * Media consumption when GHG is the pollution proxy. For CO_2_ intensity, the effect on obesity is estimated as 1.44 + 0.06 * Media consumption. When individuals do not use the Internet, i.e., Media= 0, the effect of pollution is limited at 0.13 (GHG) and 1.44 (CO_2_ intensity). In other words, assuming no media consumption, the effect of a 1% pollution increase raises the prevalence of obesity by 0.1.31% and 1.44%, respectively. When all of the adult population in a country uses the Internet, however, the effect of a 1% increase in GHG emissions on obesity is 0.161 (i.e., 0.131 + 0.03 * 1), whereas the effect of a 1% increase in the CO_2_ intensity is magnified at 1.5 (i.e., 1.44 + 0.06 * 1). Compared to the slope coefficients in M(1) − M(2), the slopes of pollution are significantly higher, and their significance is also increased by the addition of the interaction factor. Consequently, because of the interaction factor, the slope coefficients between pollution and obesity are on one hand higher and on the other hand different for the different media consumption levels.

Additionally, both specifications, M(3) and M(4), show a significant autoregressive coefficient that is, however, impacted differently by the addition of the interaction factor. Thus, when GHG proxies for pollution in M(3), the inclusion of the interaction actor decreases the magnitude of the autoregressive coefficient to 0.20, whereas in M(4) the point estimate for the auto-regressive coefficient is increased beyond the original boundaries to 0.536.

### 3.3. Further Robustness Checks

Table 7 reports the results of the System GMM estimates for alternative regressor mixes in Equation (4). The main results are still robust to the inclusion of extra variables. Alternative estimations include GDP per capita, trade openness, and government consumption as additional controls.

All specifications show a positive and significant autoregressive coefficient that has the highest magnitude when GDP per capita is not used as a control, implying that economic development does carry a large amount of information related to obesity. As can be seen, the lower bound is equal to 0.19, whereas the upper bound is 0.63.

Most importantly, the Sys-GMM parameters are always positive and significant at 1% for the two measures of pollution; however, the magnitude is higher throughout model specifications when the CO_2_ intensity factor is used as a proxy.

The interaction factor is always positive and significant; its significance, as well as its magnitude, is higher when used with the CO_2_ intensity proxy for pollution. The use of media (Internet) is by itself a statistically significant factor for obesity at 10%. Moreover, its inclusion in the mix of explanatory variables as an independent factor increases the impact of pollution (i.e., GHG) on obesity from 0.09 to 0.14, whereas its inclusion within the interaction factor also increases the impact of pollution on obesity, reaching 0.13.

Further expanding the control group by means of the addition of the government consumption factor increases the impact of pollution to 0.138, while maintaining its statistical significance at 1%.

The GovCons variable is by itself a significant impact factor for obesity, with a point estimate for its coefficient of 0.117, significant at 10%.

## 4. Discussion

The findings for the seven Sys-GMM alternative model specifications confirm a priori assumptions, namely that (i) a higher prevalence of obesity in the previous period contributes to a higher prevalence of obesity in the current period, (ii) pollution (all proxies) is the main factor that increases obesity prevalence in the sample of countries in this study, and (iii) media consumption is an independent driver for obesity; moreover, the combined effect of media consumption and pollution on obesity is positive and statistically significant, and also contributes to increasing the magnitude of the pollution impact.

The first line of results agree with previous findings that document the persistent effect of obesity ([70]). Moreover, these findings support the view of the World Obesity Federation as expressed in [71], in that obesity is a chronic, relapsing, progressive disease process and, consequently, they contribute to highlighting that immediate action is needed to prevent and control this worldwide epidemic.

Furthermore, our findings document the positive association between pollution and obesity, with the slope coefficient of the pollution factor remaining robust across different model specifications. In this respect, current research findings are in line with previous studies that conclude that pollution is a driver of obesity (i.e., [22,24]), and thus help to strengthen the evidence that there is a positive association between exposure to pollution and the risk of being overweight ([72,73]). Moreover, current findings resonate on the one hand with [25,26] in that the link between pollution and obesity can be explained by behavioral channels such as physical inactivity or sedentarism and, on the other hand, with [27], in that the link can also be explained by physical channels, such as through the thyroid function parameters. These results further complement the findings of [27], highlighting the importance of public health policy measures to reduce air pollution.

Moreover, we confirm that media consumption (as proxied by the relative number of Internet users) is positively linked to obesity, providing updated evidence to support the findings of [34,74,75,76], among others. This result is also in line with the conclusions of [28,29,77,78] that indicate that a sedentary lifestyle, concurrent snacking behavior, and reduced sleep duration can act as transmission channels between media consumption and obesity. The current findings can thus be explained by these previously documented links and resonate with the conclusion of [33] that the use of technology for entertainment both reinforces and promotes physical inactivity. We thus additionally argue that the usage of media most often replaces time spent engaging in physical activity, which can also be an explanatory factor for the current results, supported by a recent WHO study ([79]). This potential transmission channel is reinforced by [80] who showed that the amount of time teenagers spend on social media has increased, from 4.4 h per week in 2007 to 11.1 h in 2011.

Additionally, results for most specifications (except for M(1)) show no statistically significant relationship between economic development, as reflected by GDP per capita, and the prevalence of obesity, deviating from previous evidence on the positive association between low socioeconomic status and unhealthy body weight. This discrepancy might be explained, on the one hand, by the data sample used in our analysis. As such, due to the (un)availability of data, our sample is biased toward high-income and upper-middle-income countries. Moreover, this study uses national-level data, as opposed to individual-level data. Furthermore, it has been shown that the positive association between economic development and obesity is mitigated by the education level of the population, being absent or reduced for higher levels of education ([81]). In turn, education is closely linked to economic development ([82]), which in turn, given the sample bias in this study, can explain the current findings. Furthermore, [83] confirmed that GDP is positively related to BMI for levels up to approximately USD 3000 per capita, with no significant relationships beyond these levels. By comparison, our data sample reports a mean value of USD 24,000 per capita, well into the no-relationship zone. Consequently, given that our sample of countries mostly includes rich countries, and in light of previous empirical evidence that positively links education and economic growth ([82,84,85]), we conclude that a possible reason that the current results deviate from previous findings is that they are sample-specific.

On the other hand, the factor corresponding to economic development loses its statistical significance when CO_2_ intensity is introduced as a proxy for pollution or when the interaction factor is employed. This in turn suggests that CO_2_ intensity and the number of Internet users contain similar information with the economic development variable, which is plausible and in line with [86], who acknowledged the use of fossil fuels in various activities tied to economic development, which is subsequently driving carbon emissions. The correlation coefficient between media consumption and GDP (i.e., 0.56) also provided a preliminary indication in this respect. As such, the contradiction of previous findings may occur due to the additional variables employed in this study that carry more relevant information for obesity than the income factor.

Finally, the results indicate that the general government final consumption expenditure (% of GDP) is positively associated with obesity. The ratio of government consumption expenditure to gross domestic product is an indicator of government size [87], employed as a production factor in the production function [88]. Its positive impact on obesity in a model that also includes the autoregressive term, pollution (GHG), GDP per capita, and the interaction factor between pollution and media consumption deviates from the results of [89], who encountered a positive relationship between government expenditure and welfare, as represented by increased life expectancy in Pakistan, and from the results of [90], who documented a positive relationship between public expenditure on health care and health status in Lesotho. However, this can on one hand be explained by the significant difference in mean income level between the samples employed in the two studies and the current research. Additionally, this discrepancy could be the result of using different indicators for public consumption, as this study employs aggregate data on government consumption expenditure, whereas the other studies employ public expenditure on health care. The positive contribution of public expenditure to obesity found in this study can reflect indirect effects operating through the impact of government spending on pollution and/or media consumption and their subsequent positive impact on obesity.

## 5. Conclusions

Over 99% of the world’s population is exposed to air pollution levels that exceed the WHO guidelines, which in turn leads to various health-related negative outcomes.

Nonetheless, despite the recognized role of pollution in promoting unhealthy body weight, including obesity, the empirical evidence remains scarce. The aim of this paper is thus to explore the association between pollution and obesity, two paramount indicators for global population health, both responsible for various health problems, premature deaths, and significant economic costs. Drawing on unbalanced panel data for 2000–2015 and employing robust dynamic panel Sys-GMM estimators, this paper also documents trends in the main variables of interest, along with relationships between pollution, obesity, and a relevant group of control variables.

This study documents channels through which pollution impacts obesity, including the roles played by socio-economic development, government consumption, trade openness, media consumption, and the interaction between pollution and media consumption. The evidence suggests that pollution and media consumption are significant drivers for obesity. The effect of pollution is statistically significant and positive across all model specifications, but the positive effect is more robust when CO_2_ intensity is employed as a proxy for pollution than when the factor representing total GHG emissions plays the same role. Moreover, the introduction of an interaction factor representing the concurrent impact of pollution and media consumption in dynamic panel regressions increases the magnitude of the pollution impact, while also acting as a significant driver for obesity by itself. Other findings indicate that government consumption also contributes to increased obesity, whereas media consumption and CO_2_ intensity account for a large part of the association between socioeconomic status and obesity.

Two important policy implications emerge from the current results. First, countries should continue their efforts to mitigate pollution levels and meet the increasingly conservative targets established by international agreements, such as the Paris agreements and the European Green Deal. Policymakers should closely monitor whether countries are on the right track to achieve low-carbon policy targets and also intervene in a timely manner to impose stricter targets. Second, these efforts should be complemented by targeted policies and campaigns aiming to educate the population regarding the importance of physical exercise and healthy nutrition, and the negative consequences of too much and/or unhealthy media consumption. We argue that the synergy between these two approaches would contribute to attaining both sustainable low-carbon development and the sustainable reduction and prevention of obesity.

However, as is the case with most studies, the current findings have to be seen in the light of some limitations. First, due to the unavailability of data on the prevalence of obesity in most world countries, this study draws on a rather narrow and unbalanced panel of countries, which in turn imposes restrictions on statistical procedures and hinders the possibility to generalize the results. Furthermore, although the estimates of the assessed links are robust, the mechanisms are not specifically tested, being offered as potential transmission channels. Second, it should also be acknowledged that the current results emerge from country-level factors and are thus unable to capture potential distinct relationships that might characterize different categories of the population, delineated by socio-economic status or behavioral traits. Consequently, the current findings need to be supplemented by longitudinal human studies aimed at further documenting the effects of air pollution and media consumption on the development of obesity through uncovering specific transmission channels.

## Figures and Tables

**Table 1:** Variable description.

Variable Abbreviation	Variable Code (World Bank WDI Database)	Variable Description
OBES	HF.STA.OB18.ZS	Prevalence of obesity, BMI > 30 (% of population 18+) indicates the percentage of population aged 18 and older with a body mass index (BMI) above 30
GHG	EN.ATM.GHGT.KT.CE	Total greenhouse gas emissions (kt of CO_2_ equivalent)
CO_2_ intensity	EN.ATM.CO2E.EG.ZS	CO_2_ intensity (kg per kg of oil equivalent energy use) is the ratio of carbon dioxide emitted per unit of energy, or the amount of carbon dioxide emitted as a result of using one unit of energy in production.
Media	IT.NET.USER.ZS	Individuals using the Internet (% of population). As per the World Bank’s definition, individuals who have utilized the Internet (from any location) in the last three months are included in the indicator. The Internet can be accessed via a computer, mobile phone, personal digital assistant, gaming machine, digital television, and other devices.
GDP	NY.GDP.PCAP.KD	GDP per capita (constant 2015 USD)
Gov Cons	NE.CON.GOVT.ZS	General government final consumption expenditure (% of GDP)
TradeOpen	NE.TRD.GNFS.ZS	Trade openness is the sum of exports and imports of goods a services measured as a share of gross domestic product (% of GDP)

**Table 2:** The sample.

Income Level *	Countries
High-income	Austria, Belgium, Croatia, Czech Republic, Denmark, Estonia, Finland, France, Greece, Hungary, Ireland, Italy, Latvia, Netherlands, Norway, Poland, Portugal, Slovak Republic, Slovenia, Spain, Sweden, United Kingdom
Upper middle income	Bulgaria, Georgia, Iran, Russian Federation, Turkey
Low income	Nepal

* Countries are classified by World Bank income levels.

**Table 3:** Descriptive statistics.

Variable	Mean	Standard Deviation	Min	Max
Global panel				
OBES	16.11	5.79	1.70	34.20
GHG	457,431.83	763,866.04	11,170.00	2,566,170.00
CO_2_ intensity	2.21	0.62	0.29	3.49
Media	48.20	27.82	0.38	96.81
GDP	24,163.88	17,353.17	585.41	74,355.52
GovCons	18.97	4.06	7.90	26.08
TradeOpen	88.38	40.22	37.92	215.16

Source: Estimation results.

**Table 4:** The correlogram of the variables.

	GDP	CO_2_ Intensity	GHG	OBES	TradeOpen	GovCons	Media
GDP	1.00						
CO_2_ intensity	−0.18	1.00					
GHG	−0.37	0.14	1.00				
OBES	−0.26	0.27	0.38	1.00			
TradeOpen	0.21	0.05	−0.43	0.10	1.00		
GovCons	0.61	−0.16	−0.18	0.03	0.21	1.00	
Media	0.56	−0.24	−0.29	0.26	0.41	0.64	1.00

**Table 5:** One-step system GMM estimates.

Dependent Variable: Obesity	M(1)	M(2)
Independent Variables	Estimate	Estimate
Obesity (−1)	0.33 *** (0.03)	0.44 * (0.24)
GDP	0.08 * (0.01)	0.10 (0.11)
GHG	0.09 *** (0.01)	
CO_2_ intensity		0.92 *** (0.33)
Hansen/Sargan J-test (*p*-value)	0.39	0.80
AR1 test (*p*-value)	0.29	0.09
AR2 test (*p*-value)	0.28	0.32
Wald test for coefficients (*p*-value)	0.00	0.00

Standard errors are reported in parentheses; * significant at 10%; *** significant at 1%. AR1 and AR2 represent the Arellano–Bond tests for first- and second-order autocorrelation in the idiosyncratic errors of the first-differenced equation. Instruments are collapsed; robust inference is performed in the summary.

**Table 6:** Estimation results: Alternative pollution factor and the addition of the interaction actor.

**Panel A. Dependent Variable: Obesity**	**M(3)**
**Independent Variables**	**Estimate**
Obes(−1)	0.20 *** (0.05)
GDP	0.00 (0.02)
GHG	0.13 *** (0.02)
GHG * Media	0.03 ** (0.00)
Hansen/Sargan J-test (*p*-value)	0.87
AR1 test (*p*-value)	0.27
AR2 test (*p*-value)	0.31
Wald test for coefficients (*p*-value)	0.00
**Panel B. Dependent variable: Obesity**	**M(4)**
**Independent variables**	**Estimate**
Obes(−1)	0.54 *** (0.07)
GDP	0.09 (0.05)
CO_2_ Intensity	1.45 *** (0.33)
CO_2_ intensity * Media	0.06 *** (0.02)
Hansen/Sargan J-test (*p*-value)	0.92
AR1 test (*p*-value)	0.37
AR2 test (*p*-value)	0.30
Wald test for coefficients (*p*-value)	0.00

** significant at 5%; *** significant at 1%. Instruments are collapsed; robust inference is performed in the summary.

**Table 7:** Robustness checks: effect of alternative mix of control variables on obesity. Results from one-step system-GMM dynamic panel estimations.

Dependent Variable: Obesity	M(5)	M(6)	M(7)
Independent Variables	Estimate	Estimate	Estimate
Obes(−1)	0.27 *** (0.05)	0.63 *** (0.11)	0.19 *** (0.02)
GDP	0.02 (0.03)		0.01 (0.03)
GHG	0.14 *** (0.02)		0.14 *** (0.04)
CO_2_ intensity		0.50 *** (0.07)	
Media	0.009 * (0.00)		
Pollution (GHG/CO_2_ intensity) * Media		0.06 *** (0.00)	0.01 * (0.00)
TradeOpen		−0.09 (0.01)	
GovCons			0.12 * (0.07)
Hansen/Sargan J-test (*p*-value)	0.70	0.91	0.83
AR1 test (*p*-value)	0.34	0.47	0.33
AR2 test (*p*-value)	0.27	0.23	0.21
Wald test for coefficients (*p*-value)	0.00	0.00	0.00

* Indicates significance at 10% level, respectively. *** Indicates significance at 1% level, respectively. Instruments are collapsed; robust inference is performed in the summary.

## Data Availability

Data employed in this study are publicly available from the World Bank Indicators database.
